# Management of Post-Traumatic Dental Care Anxiety in Pediatric Dental Practice—A Clinical Study

**DOI:** 10.3390/children9081146

**Published:** 2022-07-29

**Authors:** Twana Othman Hussein, Damla Akşit-Bıçak

**Affiliations:** 1 Department of Paediatric Dentistry, Faculty of Dentistry, Near East University, Mersin 10, Nicosia 99138, Turkey; twana201@yahoo.com; 2 Department of Pediatric Dentistry, Faculty of Dentistry, Final International University, Mersin 10, Nicosia 99010, Turkey

**Keywords:** behavior, children, general anesthesia, pediatric dentistry, post-traumatic dental care anxiety

## Abstract

Background: It is important to avoid giving children traumatic dental experiences that induce post-traumatic dental care anxiety (PTDA) in clinical dental practices. The aim of this study was to evaluate whether non-pharmacological behavior management procedures can effectively reduce the use of pharmacological behavior management in children who have PTDA and are referred for regular dental treatments under general anesthesia (GA) and sedation. Methods: This clinical study consisted of two groups. The treatment group involved 20 healthy children aged 4–14 with PTDA and also those referred by other institutions for dental treatment with/without GA. The control group was sampled retrospectively from the patient records and involved 20 healthy uncooperative children aged 4–14 with PTDA who had been treated under GA. Results: The number of multiple appointments was significantly higher in the treatment group than in the control group. Only 25% of children in the treatment group underwent GA and the rest (75%) were managed with non-pharmacological management techniques. Nine (60%) children who were treated with minimally invasive techniques did not require GA. Conclusions: It is important to treat children as much as possible without causing PTDA by using appropriate behavior management techniques. This study emphasizes the usefulness of thoroughly employing non-pharmacological behavior management methods before directing a child with PTDA for dental treatment under pharmacological behavior management, which can prevent the over-utilization of sedation and GA.

## 1. Introduction

The terms “phobia”, “anxiety”, and “fear” often appear in dentistry-related literature. Fear is often a mild, age-related, transitory emotional state with a specific focus or stimulus. Moreover, an extreme, persistent form of fear is defined as a phobia. However, anxiety is an aversive emotional state without a specific focus or stimulus [1]. Dental anxiety, on the other hand, is defined as the patient’s response to stress that occurs specifically in dental conditions and is multifactorial [1,2]. During childhood, a child’s dental anxiety is attributed to factors such as the child’s traumatic and negative dental experiences, mostly related to painful restorative treatments, including vibrational sensations, the sounds and sights of dental drills, local anesthetic injections, extractions, as well as other factors, such as personal characteristics, parental dental fear, age, and gender. All these conditions lead to behavior management problems and dental treatment avoidance, causing poor oral health, dental pathologies, and an increase in the usage of pharmacological dental behavior management, such as general anesthesia (GA) [1,3,4,5,6,7,8,9]. Therefore, dentists should be cognizant of the fact that the first dental experience of a child usually determines their future willingness to undergo dental treatments. Thus, it is important to avoid giving children traumatic dental experiences that induce post-traumatic dental care anxiety (PTDA) in clinical practices [10,11]. Practical communication dexterity and connection with the patient are essential to provide painless dental treatment to pediatric patients in order to cope with dental fear and anxiety at an early age. Painless dental treatment sessions will reduce pre-existing anxiety and increase treatment adherence [12].

To establish successful dental treatments, the dentist’s attitude toward the child patient and the use of appropriate behavior management skills are of vital importance [7]. Behavior management requires communication, kindness, coaching, tolerance, flexibility, and engaged listening. The dental team can relieve fear and anxiety via behavior management approaches, teaching correct behavioral approaches, and coaching the child to be cooperative, calm, and compliant in the dental environment [13,14,15].

In order to prevent and/or cope with PTDA, the contribution and consciousness of parents are needed as well. Parents can be given education and advice on certain issues. Parents should understand the feelings of their children and should support them. Moreover, they should not transfer their own dental experiences to their children before bringing them to the dental appointment. At the same time, parents should be informed about mobile applications demonstrating to children how various dental treatments are performed. Furthermore, it can be advised that children can watch cartoons and/or play games about oral and dental health before coming to the dental clinic [10]. Consulting with a specialist who is educated and experienced in the dental treatments of children is also important. Various obstacles determine dental practitioners’ abilities to enforce behavior management procedures in regular clinical settings [16,17,18]. If practitioners lack behavioral management skills, the use of pharmacological dental behavior management, such as GA, has shown a significant increase in pediatric dental treatments [8,9].

Practice-based investigations have also revealed a significant escalation in the use of GA by general dental practitioners and pediatric dentists. As a result of busy schedules and parental attitudes, it is sometimes difficult for the practitioner to either refuse that type of treatment or even wish to spend the time using other techniques to avoid GA. Meanwhile, according to recent evidence, most pediatric patients can be treated in normal dental settings by establishing a proper connection with the parent and patient, and by relying on suitable behavior management approaches [19,20,21,22,23,24]. Thus, the present study was conducted to determine whether appropriate non-pharmacological behavior management procedures can effectively reduce the use of pharmacological behavior management in children who have dental anxiety because of previous dental treatments and who are referred for regular dental treatments under general anesthesia and sedation. Thus, it was hypothesized that for children with PTDA, encouraging them to cooperate via more than one appointment, and with the use of appropriate non-pharmacological behavior management techniques and minimally invasive approaches, could be effective to conduct dental treatments without the use of GA or sedation. The null hypothesis was that there was no association between methods of intervention and the outcome (whether pharmaceutical or non-pharmaceutical).

## 2. Materials and Methods

### 2.1. Ethical Considerations

Ethical approval was obtained from the Near East University Clinical Research Ethics Committee (NEU/2022/100-1503). In addition, the ethical standards of the Declaration of Helsinki were followed. Informed written consent was obtained from the parents for their children’s participation in this study.

### 2.2. Participant Recruitment

This study was carried out at the Near East University, Faculty of Dentistry, Pediatric Dentistry Department. For the sample size calculation, G*Power (Version 3.1.9.4) (Heinrich Heine University, Dusseldorf, Germany) for Mac software was used. 

Based on similar study [22] in the literature and according to the statistical evaluation with 80% statistical power and a degree of precision (d) of 0.51, it was determined that 20 patients should be involved in each group within the limitations of this study.

The study consisted of two groups. The treatment group was sampled prospectively and involved 20 children with PTDA who had been admitted to the Near East University Department of Pediatric Dentistry for dental treatment, as those children were considered definitely uncooperative and had previous traumatic dental treatment experiences. On the other hand, the control group was sampled retrospectively from the patient records and involved 20 healthy uncooperative children aged 4–14 with PTDA who had been treated under GA. All information belonging to the control group was obtained from the archive for those patients.

### 2.3. Inclusion Criteria

The inclusion criteria for the treatment group were: Children between the ages of 4 and 14 with PTDA who exhibited negative cooperation, healthy children, children with dental treatment needs, children with PTDA who had been referred from another institution to the Near East University Department of Pediatric Dentistry for dental treatment under GA, and uncooperative patients who attended or were referred to the Near East University Department of Pediatric Dentistry who had PTDA. The inclusion criteria for the control group were: children between the ages of 4 and 14 with PTDA who had been treated under GA as well as healthy children.

### 2.4. Exclusion Criteria

The exclusion criteria for both groups were: cooperative children, children who were uncooperative for reasons other than previous dental treatment, children without dental treatment needs, children with systemic diseases, and children lacking cooperation abilities.

### 2.5. Medical and Dental Anamnesis

In the first examination, the parents were asked to give information, including the patient’s name, age, gender, presence or absence of any systemic diseases and medications used (if applicable), presence of allergies, the reason for presenting to the dental clinic, and the number of previous dentists, which were recorded on patient forms. The parenting styles of the mothers or fathers in the treatment group were observed by the dental practitioner during the appointments (depressed, normal, overanxious) [25] and recorded on the patient forms.

In addition, the reasons for the children being uncooperative, including dental treatments that caused PTDA in both groups, were questioned and recorded. The dentists’ behaviors, fear transferred from mothers and siblings, previous medical treatments, dental syringes or fear of dental anesthesia applications, dental drillings, dental instruments, restorative treatments, root canal treatments, and extractions might have caused negative experiences mostly related to pain, uncooperativeness, and PTDA [1,3,4,5,6,7,8,9]. According to the dental history received from the parents, children who approached dental treatments positively and cooperatively in the dental chair at the beginning but became uncooperative for one or more of the reasons mentioned above during dental treatments were diagnosed as having PTDA.

### 2.6. Oral Examination

Children were examined on a dental chair and the teeth surfaces were evaluated with the help of a dental explorer and dental mirror after drying and under appropriate lighting. DMFT/S values for permanent teeth and DMFT/S values for primary teeth (D,d: decayed, M,m: missing, F,f: filled, and T,t: teeth) were assessed in line with World Health Organization standards [26].

### 2.7. Dental Treatments

The individual behaviors of each child in the treatment were evaluated during the first and subsequent appointments using the Frankl Behavior Scale [27]. Changes in the Frankl score from the first to last visits were evaluated for all participating children. The Frankl scale categorizes the child’s behavior in the dental clinic into four categories: rating 1 (definitely negative), rating 2 (negative), rating 3 (positive), and rating 4 (definitely positive) [27]. Ratings 1 and 2 indicate negative cooperation in the dental clinic.

Furthermore, the number of appointments attempted for the patient to adapt, non-pharmacological behavior management techniques used, and dental treatments were recorded for each appointment for children in the treatment group. In between each appointment, a period of 7 to 10 days of rest was allowed in order to enable the child to adapt. In addition, during this period, the families were given instructions for home support, such as watching videos of cooperative children receiving dental treatment and providing positive reinforcement from parents. Various non-pharmacological dental behavior management techniques were used during multiple appointments to enable the children to adapt to the dental clinic and cooperate. Tell–show–do, modeling, distraction (video, virtual reality, music, videogames), dentist controlled by the child, verbal communication, systemic desensitization, positive reinforcement, and relaxation techniques were some of the techniques used. Finally, the patients who became cooperative and those who did not cooperate with non-pharmacological behavior management techniques (and treated under GA) were recorded. Moreover, an insulin syringe was used for patients who needed local anesthesia [23] and minimally invasive treatment approaches were conducted. Among the treatment group, all oral examinations and dental treatments were conducted by the same pediatric dentistry Ph.D. student.

### 2.8. Statistical Analysis

Data entry was performed using an Excel spreadsheet and statistical analysis was then performed using the SPSS program, version 24 (IBM SPSS Statistical Package for the Social Sciences) (Chicago, Illinois, USA). Before the data entry and analysis, the questions of the study were coded. The data, presented in tabular form, show the frequency and relative frequency distributions of the different variables among both groups. Chi-square tests were used to compare the absolute data between these two groups of patients (treatment and control) concerning other variables. *p* values of 0.05 were used as cut-off points for the significance of statistical tests.

## 3. Results

A total of 40 children with PTDA (15 girls and 25 boys) between the ages of 4 and 14 years old were included in the study. The mean age in the control group was 7.10 years, with a standard deviation of 2.83. The mean age in the treatment group was 8.15 years, with a standard deviation of 3.03. There were no age differences between these two groups, while about two-thirds of the control group were male and only one-third in the treatment group (Table 1).

The number of dentists previously seen did not differ between groups. Most of the children in the control group only had one appointment, while the number of appointments in the treatment group was between two and eight, in order to allow the children to adapt to the dental treatments. Resultantly, the outcomes were significantly different as all patients in the control group were managed by GA while only 25% of the treatment group underwent GA; the rest (75%) were managed with non-pharmacological management techniques (Table 2).

Each patient in this study had a history of PTDA due to different dental treatments. There were no statistically significant differences between the applied dental treatments that caused PTDA between groups, as shown in Table 3.

The mean caries index scores did not differ according to gender, but a higher value was detected among children younger than 6 years of age, and among the control group when compared with the treatment group (*p* < 0.05) (Table 4).

Table 5 shows the relationship between the use of minimally invasive treatment techniques and the use of an insulin syringe with successful non-pharmacological interventions or GA. Nine (60%) children who were treated with minimally invasive techniques did not require GA. This result was statistically significant (*p* < 0.05). However, insulin syringe usage and the management of children with non-pharmacological interventions or GA did not differ and were non-significant statistically (*p* > 0.05) (Table 5).

The number of appointments, the dental treatments conducted in each appointment until the children cooperated, and the results for the treatment group can be seen in Table 6.

Table 7 shows the relationship between parenting style types and Frankl score changes from the first to last appointments. The parenting style did not affect changes in the Frankl score. For the majority of both the overanxious and normal parenting styles, a rating change from 1 to 4 can be seen. The results of the study showed that 70% of patients with PTDA had a parent with an overanxious parenting style, 25% had a parent with a normal parenting style, and 5% had a parent with a depressed parenting style (Table 7).

The Frankl Behavior Rating Scale changes in the treatment and control groups from the first to last appointments are presented in Table 8. There were statistically significant dissimilarities between the first appointment and the last appointment on the Frankl scale. It is clear that, after using behavior management techniques, there was a change in the Frankl rating scores from 1 to 3 or from 1 to 4 in the majority of cases (75%). Thirteen children developed to Frankl 4, and 2 developed to Frankl 3. Five children stayed as Frankl 1 (Table 8).

## 4. Discussion

This study aimed to determine whether various behavioral guidance techniques could effectively prevent the need for general anesthesia and sedation administration among patients with PTDA who were definitely negative in cooperation. To the best of our knowledge, this study is one of the first studies to examine the management of post-traumatic dental care anxiety in pediatric dental practice. In [28], the authors showed that girls are more dentally-anxious than boys. Yakar et al. [29] reported high dental anxiety scores among women. Yuwannisa et al. [30] reported that age and gender play a role among child patients regarding dental anxiety. Younger girls were found to have higher anxiety than older ones. Silveira et al. [31] and Fayad et al. [32] reported that dental fear and anxiety are more prevalent among females and younger patients. However, in the current study, most of the children in the control group who were treated under general anesthesia were boys, and there were no age differences between these two groups. Furthermore, the number of dentists previously seen did not differ between groups. In addition, most of the children in the control group only had one appointment while the number of appointments in the treatment group was between two and eight in order for the children to adapt to the dental treatments. It is obvious that increasing the number of appointments has a positive effect on a child’s cooperation. Before giving a general anesthesia indication, offering pediatric patients the opportunity to become familiar with the clinical environment and to decide a treatment plan according to each patient’s own intraoral situation is extremely important in clinical practice.

Negative dental treatment experiences were linked with increased dental fear among young adults [3,6]. Extractions, drilling, restorative treatments, fear transferred from mothers or siblings, applications of local anesthesia, root canal treatments, and previous dental treatments can potentially trigger dental fear and anxiety [33]. Roopnarine et al. [34] reported a high prevalence of dental anxiety due to local anesthesia applications and dental extractions. In another study [35], the highest fear and anxiety were observed among adolescents who had a tooth extracted at their last appointment. De Jongh et al. [36] reported that horrific experiences during dental treatments and traumatic medical experiences were the most common causes of dental anxiety. Abrahamsson et al. [37] showed that the occurrence of dental fear was related to traumatic dental care experiences and personal characteristics in addition to the dentists’ behavior. Stenebrand et al. [38] reported that previous pain during dental treatments can cause high dental anxiety among 15-year-old patients. Other studies [35,39] have also shown that younger children and children with negative dental experiences exhibited higher dental anxiety. In the current study, the primary causes of PTDA among the control group were dental anesthesia applications, restorative treatments, followed by drilling, and the dentist’s behavior, whereas among the treatment group, they were restorative treatments, followed by medical treatments, the dentist’s behavior, and dental anesthesia. Contrary to the literature, dental extractions were not applied to the children before and there were no statistically significant differences in the types of previously applied dental treatments between the treatment and control groups.

The development of new carious lesions is one of the most significant risk factors for the occurrence of dental fear and anxiety. The prevention of dental caries is important in order to decrease the need for further treatments, which may cause traumatic and painful experiences. In previous research, it was shown that dental fear was greater among children aged 7–9 years who had dental caries and who experienced dental pain [40]. Silveira et al. [31] reported that dental fear was more prevalent among children who had dental caries. In line with the literature, in the current study, higher caries index scores were detected among children younger than 6 years of age and the control group when compared with the treatment group. Furthermore, in order to accomplish successful dental treatments with young children, applying painless local anesthesia [41] and the use of minimally invasive treatment approaches (silver diamine fluoride, atraumatic restorative treatments, hall technique, interim therapeutic restorations, etc.) are crucial. Moreover, new and additional types of injectors (insulin syringes) could be highly applicable for treating these children in non-pharmacological ways [23]. In the current study, 60% of children who were treated with minimally invasive techniques did not require GA.

While conducting the dental treatments of children with PTDA, the success rates of non-pharmacological dental behavior management by using multiple adaptation appointments were also examined in this study. The most striking result from this study was that 75% of children with PTDA who applied or were referred to our clinic for dental treatment under GA were handled or rehabilitated to be treated successfully in a standard dental atmosphere without operating sedatives and only using communicative guidance. However, in a previous study conducted by Aminabadi et al. [42], it was reported that 47.5% of children who were referred for dental treatment under GA were accurately handled or rehabilitated to be treated in a regular dental environment using behavior management techniques in addition to the use of conscious sedation (nitrous oxide, sedatives, or both) and restraints. The dentist or pediatric dentist should choose an efficient and appropriate treatment modality for each pediatric patient. In another study conducted by Tyrer [21], 75% of dental treatments were completed without using GA among children between the ages of 3 and 14. Consequently, the outcomes of this study may provide added support for the argument that rehabilitating pediatric patients with PTDA using only non-pharmacological behavior guidance techniques may be a practical way of relieving fear and anxiety and directing pediatric patients to be cooperative via cognitive–behavioral approaches. However, the various behavior guidance approaches must be “tailored to individual patients and practitioners” [43]. The present study results demonstrated that a relatively high ratio of “uncooperative” children with PTDA (Frankl 1) in the treatment group was successfully handled or rehabilitated by proper non-pharmacological behavioral management procedures in the traditional dental setting. Hence, our investigation reveals the influence of ability, skill, and tolerance on the practitioners’ readiness and ability to treat pediatric dental patients. The practice of pediatric dentistry requires the use of a combination of clinical dexterities, varying from simple to very complicated. More complex approaches might need an additional internship to be effectively used, and treatment under GA should be regarded as the final treatment possibility. These skills are formulated over time by practitioners who are willing to put the time and effort into treating more children. Patience is a crucial component of behavioral modification approaches.

Undoubtedly, more research is required in this area for a more in-depth understanding of the use of proper measures before arranging any specific management techniques. While non-pharmacological behavior management procedures were not as practical in children in the control group, many of those in the treatment group were retrained using behavior management approaches. In contrast, patients in the treatment group were wholly referred to as definitely negative in cooperation. Not using an alternative type of dental syringe, trusting the general dentist’s decision about the child’s cooperation, avoiding costless adaptation appointments, the demands from parents for sedation and general anesthesia, impatient parents who want all treatments done as soon as possible, and the policies of private clinics could all be causes that encourage pediatric dentists to treat children with sedation and general anesthesia. Moreover, it can be assumed that non-pharmacological behavior management techniques were correctly practiced in our treatment group before deciding to treat these children with GA. Behavior guidance is based on scientific principles. The appropriate execution of behavior guidance requires these principles to be implemented [7]. However, non-mandatory treatment with sedation and the GA of children with PTDA among pediatric dentists emphasizes that behavior guidance needs patience, communication, sympathy, flexibility, coaching, and listening dexterities. It is a clinical skill based on scientific regulations [7,43]. In addition, this result may be attributed to the significant variability in terms of the focus on behavior guidance training in pediatric dentistry curriculums and standards as well as the unpredictable attitudes and behaviors of program managers.

It is clear that, after using behavior management techniques, changes in the Frankl rating scores from 1 to 3 or from 1 to 4 occurred for the majority of cases. The discrepancy among the number of patients rehabilitated, i.e., who developed into cooperative patients (Frankl 4) between the first and last appointments, was statistically significant in the treatment group. Seventy percent of patients with PTDA had a parent with an overanxious parenting style; however, the parenting style did not affect changes in the Frankl score from the first to the last appointments. Hence, this result adds importance to our overall results by verifying that most of the treatments with GA in post-traumatic dental anxiety cases in the control group were due to the lack of ability to differentiate between correct and incorrect uncooperative patients and the absence of necessary training skills to implement behavior management techniques. Many investigations have revealed that general dentists are less likely to treat young children if there are pediatric dentists available to whom the children can be referred [15,44,45]. However, the findings imply that many pediatric dentists did not exhibit enough tolerance to follow methodical behavior management guidelines [46,47]. The current trend may cause inaccurate referrals for general anesthesia and treatment with GA and sedation, among both pediatric and general dentists [47,48]. Thus, being trained and experienced in managing children using advanced behavior management procedures, which help overcome significant obstacles in the treatment of pediatric patients, namely fear, anxiety, and behavioral issues, are essential [21,42,43,49].

Finally; the first dental examination and treatments are very important for children and are likely to determine their approaches to lifelong dental treatments. For this reason, dentists should make treatment plans in accordance with the age, treatment needs, and degree of cooperation of each child patient. It is important to treat the children as much as possible without causing trauma by using appropriate behavior management techniques. In young age groups and the presence of early childhood caries, treatment in a dental chair is more difficult and the use of pharmacological treatment approaches becomes mandatory. For this reason, it is necessary to introduce the children to the pediatric dentist at an early age, if possible, when the first primary tooth erupts, to continue with periodic controls, and to gain appropriate nutrition and oral hygiene habits through family education. This approach prevents the development of caries and prevents the formation of oral problems during early childhood. Furthermore, the use of minimally invasive approaches may also support children in experiencing more comfortable treatment sessions.

The use of pharmacological treatment approaches, such as sedation and general anesthesia with the correct indication, is extremely beneficial and can also prevent the development of further dental fear and anxiety among children. However, unnecessary sedation and GA applications should be avoided by having knowledge and education about treating children in clinical dental practices; conducting treatments that will not cause negative and traumatic dental treatment experiences; taking all measures to ensure that the child feels as little pain as possible; re-evaluating pharmacologic treatment indications among children who were referred for dental treatments under GA from another institution; and making special assessments for each child.

In this study, the number of partipants could have been higher. This may have been due to the need of a lot of time to adapt and treat pediatric patients with PTDA in the dental clinic, the low number of population living in the geographical area, the sufficient number of pediatric dentists, and the high level of parental education and consciousness. Moreover, the absence of different injection systems [41], such as the Wand System, in order to reduce injection pain can be considered as another limitation of this study.

## 5. Conclusions

In this clinical study, it was found that children who suffered from PTDA could be retrained and treated with non-pharmacological behavior management procedures. Behavioral (non-pharmaceutical) management for those children reduced the need for dental treatment under general anesthesia in three-quarters of patients between two and six appointments. Most pediatric patients can be treated in normal dental settings by establishing a proper connection with the parent and patient, and by relying on suitable behavior management approaches, such as those used in our research: multiple visits to help a child adapt to the dental clinic, desensitization techniques, positive reinforcement, relaxation methods, dentist’s control of the child, and distractions. Furthermore, the use of minimally invasive approaches, such as ITR and ART, were effective, and could be used to treat children to avoid GA and sedation. This study emphasized the usefulness of thoroughly employing non-pharmacological behavior management methods before directing a child with PTDA for dental treatment under pharmacological behavior management, which can prevent the overutilization of sedation and general anesthesia applications. Thus, this study is one of the first studies that examined the management of PTDA in pediatric dental practice; however, further studies are needed using up-to-date dental treatment modalities, behavior management techniques, and injection systems with more participants in order to support this subject and share clinical experiences. 

## Figures and Tables

**Table 1:** Age and gender distribution of the study participants.

		Control Group (*n*%)	Treatment Group (*n*%)	Total (*n*%)	*p* Value
Age	Mean ± SD	7.10 ± 2.83	8.15 ± 3.03	7.63 ± 2.94	0.26
	<6 years	5 (50%)	5 (50%)	10 (100%)	0.66
	6–10 years	13 (54.16%)	11 (45.8%)	24 (100%)	
	11–15 years	2 (33.33%)	4 (66.6%)	6 (100%)	
Gender	Male	16 (64%)	9 (36%)	25 (100%)	0.02
	Female	4 (26.66%)	11 (73.33%)	15 (100%)	
Total		20 (50%)	20 (50%)	40 (100%)	

**Table 2:** Frequency of the number of previously seen dentists and the number of appointments attempted for the patient to adapt.

		Control Group (*n*%)	Treatment Group (*n*%)	Total (*n*%)	*p* Value
Number of dentists previously seen	0–1	6 (30%)	12 (60%)	18 (45%)	0.14
	2–3	12 (60%)	6 (30%)	18 (45%)	
	More than 3	2 (10%)	2 (10%)	4 (10%)	
Number of appointments attempted for the patient to adapt	One appointment	17 (85%)	1 (5%)	18 (45%)	<0.001
	Two–six appointments	3 (15%)	19 (95%)	22 (55%)	
Results	General anesthesia	20 (100%)	5 (25%)	25 (62.5%)	<0.001
	Non-pharmaceutical behavior management	0 (0%)	15 (75%)	15 (37.5%)	
Total		20 (100%)	20 (100%)	40 (100%)	

**Table 3:** Frequency of PTDA causes during dental treatments.

Causes of PTDA during Dental Treatments	Control Group (*n*%)	Treatment Group (*n*%)	Total (*n*%)	*p* Value
Dentist’s behavior	2 (10%)	3 (15%)	5 (12.5%)	0.25
Fear transferred from mother and siblings	1 (5%)	1 (5%)	2 (5%)	
Previous medical treatments	0 (0%)	3 (15%)	3 (7.5%)	
Dental syringes or fear of dental anesthesia applications	7 (35%)	2 (10%)	9 (22.5%)	
Dental drilling and dental instruments	3 (15%)	1 (5%)	4 (10%)	
Restorative treatments	7 (35%)	8 (40%)	15 (37.5%)	
Root canal treatments	0 (0%)	1 (5%)	1 (2.5%)	
Extraction	0 (0%)	1 (5%)	1 (2.5%)	
Total	20 (100%)	20 (100%)	40 (100%)	

**Table 4:** The mean caries index scores of the study participants.

		Mean DMFT/DMFT ± SD	*p* Value
Age	<6 years	9.00 ± 3.45	0.04
	6–10 years	7.63 ± 4.03	
	11–15 years	4.00 ± 1.67	
Gender	Male	7.72 ± 3.58	0.54
	Female	6.93 ± 4.45	
Group	Control group	9.10 ± 3.68	0.01
	Treatment group	5.75 ± 3.40	
Total		7.43 ± 3.89	

**Table 5:** The relationship between the use of minimally invasive treatment techniques and the use of an insulin syringe with non-pharmacological interventions or GA.

		Behavior Management		Total	*p* Value
		Non-Pharmacological	GA		
Use of minimally invasive techniques	Yes	9 (60%)	0 (0%)	9 (45%)	0.02
	No	6 (40%)	5 (100%)	11 (55%)	
Use of insulin syringe	Yes	5 (33.3%)	0 (0%)	5 (25%)	0.14
	No	10 (66.7%)	5 (100%)	15 (75%)	
Total		15 (100%)	5 (100%)	20 (100%)	

**Table 6:** The number of appointments and the dental treatments conducted at each appointment until the children cooperated in the treatment group.

No	Age and Gender of Patients		Dental Treatments Conducted in the 1st Appointment	Dental Treatments Conducted in the 2nd Appointment	Dental Treatments Conducted in the 3rd Appointment	Dental Treatments Conducted in the 4th Appointment	More Appointments Needed/Not Needed for Cooperation	Result (Non-Pharmacological Management, or GA)
1	10 years	F	Oral examination	Composite restoration	ITR	RCT	Child became cooperative	Non-pharmacological
2	8 years	F		Extraction	-	-		
3	5 years	M		Tooth polishing	Compomer restoration	Hall technique		
4	13 years	M		Scaling and polishing	Composite restoration	Root canal treatment		
5	10 years	M		ITR, Removing space maintainer	Extraction of mobile primary tooth	Extraction of mobile primary tooth		
6	13 years	M		Glass ionomer restoration	Composite restoration	Composite Restoration		
7	7 years	M		Composite Restoration	Hall technique	ART		
8	7 years	F		ART	ART	ART	Composite restoration in the 5th appointment, child became cooperative	
9	14 years	F		Composite restoration	Composite restoration	-	Child became cooperative	
10	12 years	F		Tooth polishing	Dental scaling and polishing	Dental scaling		
11	9 years	F		ART	Extraction	Child became cooperative		
12	6 years	F		ART	ART	Compomer restoration		
13	7 years	M		Tooth polishing	RCT	RCT		
14	4 years	M		Tooth polishing	Compomer Restoration	Compomer Restoration	Hall Technique, child became cooperative	
15	5 years	M		Compomer Restoration	RCT	-	Child became cooperative	
16	5 years	M		Child refused treatment	Child refused treatment	Child refused treatment	Child refused treatment	GA
17	9 years	F						
18	6 years	F						
19	5 years	F						
20	8 years	F						

ART: atraumatic restorative treatment, ITR: interim therapeutic restoration, RCT: root canal treatment.

**Table 7:** The relationship between parenting style types and Frankl score changes from the first to last appointments.

Frankl Score Change from First to Last Appointment	Parenting Style				*p* Value
	Depressed	Normal	Overanxious	Total	
Remained in rating 1	0	2 (40%)	3 (60%)	5 (100%)	0.69
Rating 1 to rating 3	0	1 (50%)	1 (50%)	2 (100%)	
Rating 1 to rating 4	1 (5%)	2 (10%)	10 (85%)	13(100%)	
Total	1 (5%)	5 (25%)	14 (70%)	20 (100%)	

**Table 8:** Frankl Behavior Rating Scale changes in the treatment and control groups from the first to last appointments.

		Control Group	Treatment Group	*p* Value
Frankl Score changes from the first to last appointments	No difference (remained in rating 1)	20 (100%)	5 (25%)	<0.001
	From rating 1 to rating 3	0	2 (10%)	
	From rating 1 to rating 4	0	13 (65%)	
Total		20 (100%)	20 (100%)	

## Data Availability

The data that support the findings of this study are available upon request from the corresponding author.
